# The association between heart rate variability and early kidney damage: The mediating role of blood lipid levels among hypertensive patients in a single-center cohort study

**DOI:** 10.1097/MD.0000000000048197

**Published:** 2026-04-10

**Authors:** Ningning Xie, Fang Gao, Weidong Zhao, Jie Li, Jie Cao, Shaoqing Li

**Affiliations:** a Department of Laboratory, Cangzhou Central Hospital, Cangzhou City, Hebei Province, China; b Department of Endocrine, Cangzhou Central Hospital, Cangzhou City, Hebei Province, China; c Department of Obstetrics, Cangzhou Integrated Traditional Chinese and Western Medicine Hospital, Cangzhou City, Hebei Province, China; d Department of Physical Therapy, Cangzhou Central Hospital, Cangzhou City, Hebei Province, China.

**Keywords:** blood lipid levels, cohort study, early kidney damage, heart rate variability, hypertension

## Abstract

This study aimed to investigate the association between heart rate variability (HRV) and the risk of early kidney damage (EKD) among hypertensive patients, with particular emphasis on the mediating role of low-density lipoprotein cholesterol (LDL-C). This retrospective cohort study included 1250 hypertensive patients treated between 2015 and 2020 in Hebei Province, China, with follow-up data available through December 31, 2022. Participants were followed through routine clinical visits and electronic medical record review, during which renal function indicators were periodically reassessed. Baseline HRV was measured using 24-hour Holter monitoring, with the standard deviation of all normal-to-normal intervals (SDNN) as the primary HRV index. Cox proportional hazards models were used to estimate hazard ratios for EKD, adjusting for demographic, lifestyle, clinical, and laboratory factors. Exploratory mediation analysis assessed the indirect association via lipid parameters. Higher HRV was significantly associated with an increased risk of EKD. In the fully adjusted model, participants in the highest HRV quartile had approximately twice the EKD risk compared with those in the lowest quartile (hazard ratio = 2.00, 95% confidence interval: 1.85–2.15). Low-density lipoprotein cholesterol statistically explained 22.5% of the association between HRV and EKD. Sensitivity analyses, including medication adjustment, alternative HRV metrics, spline modeling, competing-risk regression, and age stratification, confirmed the robustness of the findings. Elevated HRV, measured by 24-hour Holter-derived SDNN, was associated with increased EKD risk in hypertensive patients, with LDL-C acting as a significant intermediary factor. These findings highlight the potential value of integrating autonomic function assessment and lipid monitoring in early renal risk stratification among hypertensive populations.

## 1. Introduction

Early kidney damage (EKD) is a critical global health issue, representing the early stages of chronic kidney disease (CKD), which affects an estimated 10% to 15% of adults worldwide.^[1-3]^ Chronic kidney disease contributes to substantial morbidity, mortality, and healthcare costs, often due to its progression to end-stage renal disease (ESRD) and cardiovascular complications. In China, the prevalence of CKD is reported to be approximately 10.8%, with over 119.5 million individuals affected.^[4]^ Among these, hypertension is a leading cause, identified in more than 60% of CKD patients, and is frequently associated with EKD.^[5]^ Alarmingly, EKD often goes undetected due to the lack of overt symptoms in its early stages, underscoring the need for early identification and intervention.

Heart rate variability (HRV), a widely used noninvasive measure of autonomic nervous system activity, has been recognized as a potential marker for cardiovascular and renal health. Reduced HRV indicates autonomic imbalance, characterized by heightened sympathetic and diminished parasympathetic activity, which are closely linked to hypertension, vascular dysfunction, and kidney damage.^[6,7]^ Epidemiological studies have shown that reduced HRV is associated with increased risks of CKD and progression to ESRD. For example, a cohort study of 2500 individuals found that lower HRV predicted a 40% higher risk of CKD progression over a 10-year follow-up period.^[8]^ However, the specific mechanisms underlying this association, particularly in the context of EKD, remain unclear.

Although most population studies emphasize that reduced HRV is detrimental, emerging evidence shows that in pathological states such as hypertension an excessively high standard deviation of all normal-to-normal intervals (SDNN) may also signal risk.^[9]^ Markedly elevated SDNN can reflect large sympathetic oscillations, heightened blood pressure (BP) variability and a greater burden of premature ventricular contractions, all of which impose sustained haemodynamic and inflammatory stress on the renal microvasculature.^[10]^ Observational work has even revealed J-shaped associations in which both low and high HRV confer adverse outcomes.^[11-13]^ In hypertensive cohorts, elevated SDNN has correlated with sympathetic overactivity and cardiac structural changes, suggesting that a “very-high-HRV” phenotype may capture autonomic instability rather than protective vagal tone.^[14,15]^

Dyslipidemia, particularly elevated low-density lipoprotein cholesterol (LDL-C), is a well-established contributor to vascular and renal damage. Globally, approximately 39% of adults have elevated LDL-C, with prevalence rates higher in hypertensive populations.^[16]^ In China, dyslipidemia prevalence has risen significantly, affecting over 43% of adults aged 40 and above.^[17]^ Dyslipidemia contributes to endothelial dysfunction, oxidative stress, and systemic inflammation, all of which can exacerbate kidney injury. Emerging evidence suggests that LDL-C may mediate the relationship between autonomic dysfunction (as measured by HRV) and renal damage.^[18,19]^ Despite this, studies exploring the mediating role of LDL-C in the HRV–EKD relationship, particularly in high-risk groups such as hypertensive patients, are sparse.

Hypertension, a key modifiable risk factor for EKD, affects over 1.3 billion individuals globally and is particularly prevalent in China, where 23.2% of adults are diagnosed with the condition.^[20,21]^ Given the interplay between hypertension, HRV, and lipid metabolism, understanding how these factors interact to influence EKD risk is critical. Specifically, LDL-C may serve as a modifiable target to mitigate the impact of reduced HRV on renal health, offering opportunities for early intervention in hypertensive patients.^[22]^

This study aims to investigate the association between HRV and EKD among hypertensive patients, with a particular focus on the mediating role of LDL-C. By leveraging longitudinal data from a single-center cohort, this study seeks to provide new insights into the pathways linking autonomic nervous system activity, lipid metabolism, and kidney health.

## 2. Materials and methods

### 2.1. Data source and ethical considerations

This cohort study was carried out at Cangzhou Central Hospital, China, in accordance with the Declaration of Helsinki. The study protocol received prospective approval from the hospital’s Institutional Review Board (IRB No. CZ-D-2023057684). All participants were adults (≥18 years) and provided written informed consent permitting the research use and publication of their electronic medical records, laboratory data, and 24-hour Holter monitor recordings. The data were extracted from the hospital’s clinical database and de-identified before analysis. No additional procedures or incentives were introduced beyond routine clinical care.

### 2.2. Study population

**Figure 1.**
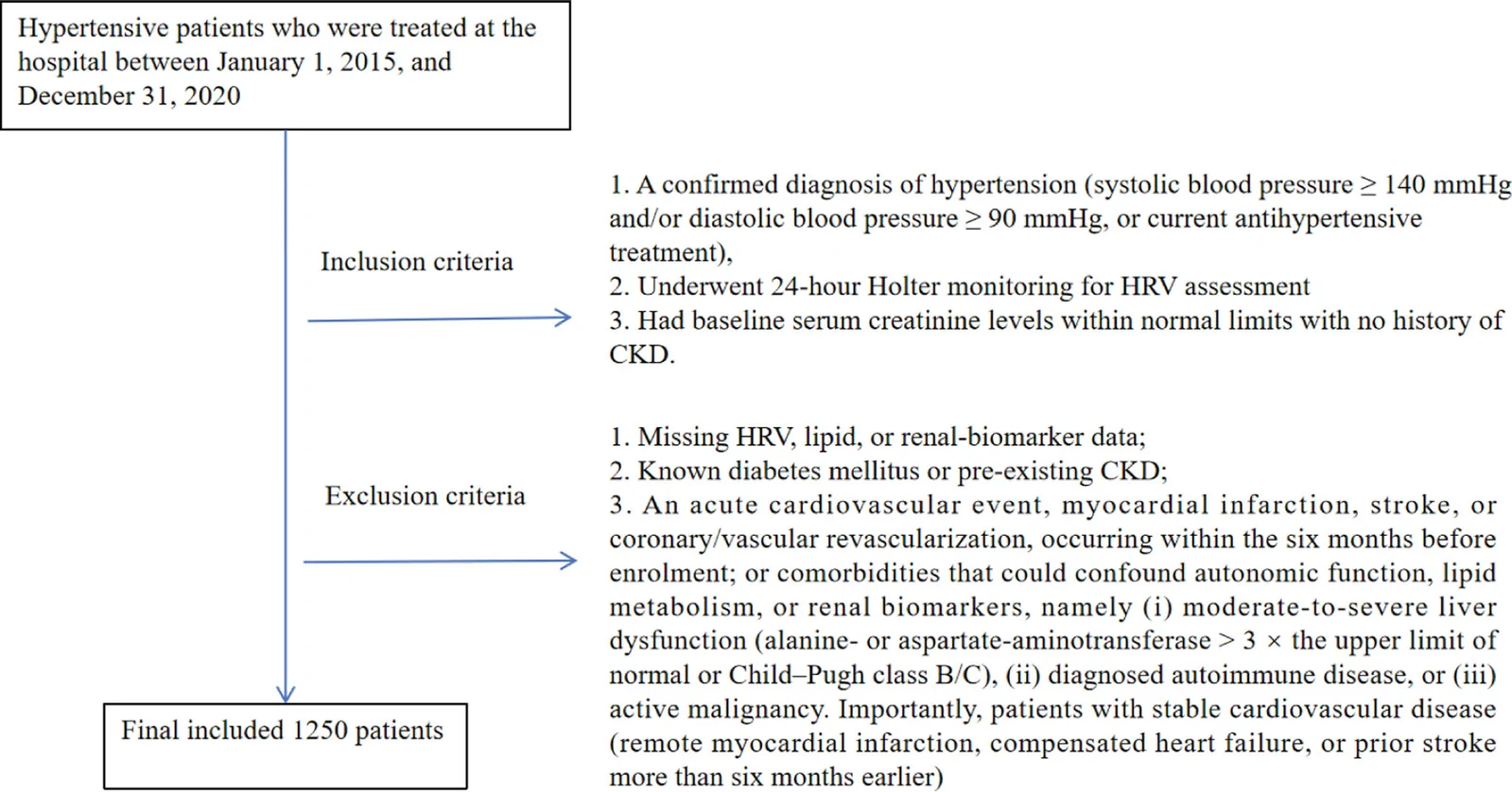
Flowchart for the inclusion and exclusion criteria of the study patients. CKD = chronic kidney disease, HRV = heart rate variability.

The cohort comprised hypertensive patients who were treated at the hospital between January 1, 2015 and December 31, 2020, with follow-up data available through December 31, 2022. Participants were eligible for inclusion if they had a confirmed diagnosis of hypertension (systolic BP ≥ 140 mm Hg and/or diastolic BP ≥ 90 mm Hg, or current antihypertensive treatment), underwent 24-hour Holter monitoring for HRV assessment, and had baseline serum creatinine levels within normal limits with no history of CKD. Individuals were excluded when any of the following applied: missing HRV, lipid, or renal-biomarker data; known diabetes mellitus or preexisting CKD; an acute cardiovascular event, myocardial infarction, stroke, or coronary/vascular revascularization, occurring within the 6 months before enrollment; or comorbidities that could confound autonomic function, lipid metabolism, or renal biomarkers, namely moderate-to-severe liver dysfunction (alanine- or aspartate-aminotransferase > 3 × the upper limit of normal or Child–Pugh class B/C), diagnosed autoimmune disease, or active malignancy. Importantly, patients with stable cardiovascular disease (CVD; remote myocardial infarction, compensated heart failure, or prior stroke more than 6 months earlier) were not excluded; their status was recorded and adjusted for as a covariate (Fig. 1). Participants were followed through routine outpatient visits and electronic medical records until December 31, 2022. Follow-up assessments typically occurred annually or when clinically indicated. Renal function indicators, including serum creatinine, estimated glomerular filtration rate (eGFR), and urinary albumin-to-creatinine ratio (UACR), were reassessed during these visits. Incident EKD was defined prospectively as the first occurrence of either UACR ≥ 30 mg/g or eGFR < 90 mL/min/1.73 m^2^ during follow-up among participants with normal baseline renal function.

### 2.3. Measurement of heart rate variability

Baseline heart-rate variability was evaluated from 24-hours Holter recordings obtained with a Meditech CardioMera 3000 system.^[23,24]^ Raw RR-interval data were automatically scanned and manually validated before analysis. Time-domain indices included the SDNN and the root-mean-square of successive differences; frequency-domain indices were low-frequency power (0.04–0.15 Hz), high-frequency power (0.15–0.40 Hz) and the low-frequency/high-frequency ratio. SDNN values are expressed in milliseconds without transformation or inversion, so a larger SDNN denotes greater beat-to-beat variability. In healthy adults, SDNN ≥ 100 milliseconds is considered normal, 50 to 99 milliseconds borderline and <50 milliseconds reduced; however, distributions in hypertensive cohorts are typically lower. Accordingly, participants were classified into quartiles based on the sample’s SDNN distribution: Q1 ≤62 milliseconds, Q2 63 to 79 milliseconds, Q3 80 to 97 milliseconds and Q4 ≥98 milliseconds. Q1 therefore represents the lowest HRV (greatest autonomic dysfunction) and served as the reference category in risk models.^[25,26]^

### 2.4. Assessment of early kidney damage

Early kidney damage was defined using UACR and eGFR. Measurements included: UACR: a UACR ≥ 30 mg/g was classified as indicative of EKD. eGFR: Calculated using the Chronic Kidney Disease Epidemiology Collaboration (CKD-EPI) equation, with a value < 90 mL/min/1.73 m^2^ indicating potential renal impairment.^[27,28]^

### 2.5. Blood lipid analysis

Fasting (≥10 hours) venous blood was drawn the morning after the Holter study. Total cholesterol (TC), triglycerides (TG), low-density lipoprotein cholesterol (LDL-C) and high-density lipoprotein cholesterol (HDL-C) were quantified by enzymatic color-metric assays on a Cobas 8000 modular analyzer (c 502 module; Roche Diagnostics GmbH, Mannheim, Germany). Reagents, calibrators and 2-level internal quality-control materials (PreciControl ClinChem Multi 1/2) were supplied by the manufacturer; calibration was performed with every reagent lot and verified daily. Inter-assay coefficients of variation during the study period were 2.3% for TC, 3.1% for TG, 2.7% for LDL-C and 2.5% for HDL-C. LDL-C was measured directly whenever TG ≥ 4.5 mmol/L and calculated by the Friedewald equation otherwise. External proficiency testing was provided quarterly by the National Center for Clinical Laboratories (Beijing), with all results within acceptable limits. Dyslipidaemia was classified according to the 2016 Chinese Guidelines for the Management of Dyslipidaemia: TC ≥ 6.2 mmol/L, TG ≥ 2.3 mmol/L, LDL-C ≥ 4.1 mmol/L or HDL-C < 1.0 mmol/L, or current lipid-lowering therapy.^[29,30]^ High-sensitivity C-reactive protein (hs-CRP) was measured using an immunoturbidimetric assay on the Cobas 8000 analyzer following manufacturer protocols. Inter-assay coefficients of variation were below 5%. Comorbidities, including CVD and diabetes status, were identified based on physician diagnoses documented in medical records, medication history, and laboratory criteria according to established clinical guidelines.

### 2.6. Covariates and confounders

To control for potential confounding factors, data were collected on demographic characteristics (age and gender), lifestyle factors (smoking, alcohol consumption, and physical activity levels), clinical variables (body mass index [BMI], BP, antihypertensive medication use, and history of CVDs), and laboratory markers, including fasting glucose and inflammatory markers such as C-reactive protein (CRP) and all blood lipid indicators. Medication data (β-blockers, statins, ACEI/ARB) were retrieved from electronic records and included in extended models.

Lifestyle factors (smoking, alcohol consumption, and physical activity) were obtained from a structured baseline questionnaire. Physical activity was assessed with the validated Chinese version of the International Physical Activity Questionnaire-Short Form (IPAQ-SF).^[31]^ Total weekly energy expenditure was calculated as metabolic-equivalent minutes per week (MET-min/wk) following the official IPAQ scoring protocol. Participants accumulating ≥ 600 MET-min/wk (roughly ≥ 150 minutes of moderate or ≥75 minutes of vigorous activity) were classified as “active”; those below this threshold were classified as “inactive.” The binary variable is presented in Table 1, and the continuous MET-min/wk variable was retained for sensitivity analyses.

**Table 1:** Baseline characteristics of participants grouped by HRV quartiles (SDNN values).

Variables	Total (N = 1250)	Q1 (N = 312)	Q2 (N = 313)	Q3 (N = 312)	Q4 (N = 313)	*P* value
Demographic characteristics						
Age (yr)	61.5 ± 9.0	59.2 ± 8.4	60.3 ± 8.6	62.5 ± 9.1	63.5 ± 9.2	<.01
Female, N (%)	670 (53.6)	140 (44.9)	155 (49.5)	185 (59.3)	190 (60.7)	.03
Residence, N (%)						<.01
Urban	800 (64.0)	200 (64.1)	210 (67.1)	195 (62.5)	195 (62.3)	
Rural	450 (36.0)	112 (35.9)	103 (32.9)	117 (37.5)	118 (37.7)	
Marital status, N (%)						.02
Married	970 (77.6)	235 (75.3)	245 (78.3)	245 (78.5)	245 (78.3)	
Unmarried	280 (22.4)	77 (24.7)	68 (21.7)	67 (21.5)	68 (21.7)	
Education level, N (%)						<.01
Primary school or below	300 (24.0)	100 (32.1)	85 (27.2)	65 (20.8)	50 (16.0)	
Middle/high school	650 (52.0)	145 (46.5)	165 (52.7)	170 (54.5)	170 (54.3)	
College or above	300 (24.0)	67 (21.5)	63 (20.1)	77 (24.7)	93 (29.7)	
Lifestyle factors						
Smoking status, N (%)						.82
Never	600 (48.0)	145 (46.5)	150 (47.9)	150 (48.1)	155 (49.5)	
Former	380 (30.4)	90 (28.8)	95 (30.4)	95 (30.4)	100 (31.9)	
Current	270 (21.6)	77 (24.7)	68 (21.7)	67 (21.5)	58 (18.5)	
Alcohol consumption, N (%)	480 (38.4)	110 (35.3)	115 (36.7)	125 (40.1)	130 (41.5)	.02
Physical activity levels, N (%)	820 (65.6)	185 (59.3)	205 (65.5)	215 (68.9)	215 (68.7)	.02
Clinical variables						
BMI (kg/m^2^)	24.1 ± 3.9	23.6 ± 3.8	23.9 ± 3.9	24.2 ± 4.0	24.8 ± 3.8	.02
Blood pressure (mm Hg)						<.01
Systolic	145.5 ± 18.7	143.0 ± 18.5	144.5 ± 18.6	147.0 ± 18.9	147.5 ± 18.8	
Diastolic	88.4 ± 12.3	87.2 ± 12.0	88.0 ± 12.2	89.3 ± 12.5	89.8 ± 12.4	
Antihypertensive use, N (%)	950 (76.0)	230 (73.7)	240 (76.7)	245 (78.5)	235 (75.1)	.08
History of CVD, N (%)	190 (15.2)	35 (11.2)	40 (12.8)	55 (17.6)	60 (19.2)	<.01
Blood lipid indicators						
Total cholesterol (TC, mmol/L)	4.9 ± 1.2	4.7 ± 1.1	4.8 ± 1.2	5.0 ± 1.2	5.2 ± 1.3	<.01
Triglycerides (TG, mmol/L)	1.8 ± 0.8	1.6 ± 0.7	1.7 ± 0.8	1.9 ± 0.8	2.0 ± 0.9	.01
LDL-C (mmol/L)	2.5 ± 0.7	2.3 ± 0.6	2.4 ± 0.7	2.6 ± 0.8	2.7 ± 0.8	<.01
HDL-C (mmol/L)	1.3 ± 0.4	1.4 ± 0.4	1.3 ± 0.4	1.2 ± 0.4	1.2 ± 0.4	.03

Data are presented as mean ± SD or N (%). The history of CVD includes prior but clinically stable coronary artery disease, heart failure, or stroke; patients with an acute cardiovascular event within the 6 months before enrollment were excluded. Physical activity assessed with the IPAQ-SF; “active” denotes ≥600 MET-min/wk.

BMI = body mass index, CVD = cardiovascular disease, HDL-C = high-density lipoprotein cholesterol, HRV = heart rate variability, IPAQ-SF = International Physical Activity Questionnaire-Short Form, LDL-C = low-density lipoprotein cholesterol, MET = metabolic equivalent of task, SD = standard deviation, SDNN = standard deviation of normal-to-normal intervals, TC = total cholesterol, TG = triglycerides.

### 2.7. Statistical analysis

Descriptive statistics were used to summarize baseline characteristics across HRV quartiles, with continuous variables expressed as means ± standard deviations or medians with interquartile ranges, and categorical variables as frequencies and percentages. Comparisons among quartiles were conducted using ANOVA or Kruskal–Wallis tests for continuous variables and chi-square tests for categorical variables.^[32]^ The primary outcome, the incidence of EKD, was evaluated using Cox proportional hazards regression to calculate hazard ratios (HRs) with 95% confidence intervals (CIs) for each HRV quartile (based on SDNN values), using the lowest quartile (Q1) as the reference. Three models were constructed to progressively address potential confounding factors: model I was unadjusted (crude model); model II included adjustments for age and gender; and model III incorporated additional adjustments for demographic, lifestyle, and clinical variables.^[33]^ These variables included residence (urban/rural), marital status, education level, BMI, smoking status, alcohol consumption, physical activity levels, antihypertensive medication use, systolic and diastolic BP, and history of CVD. Blood lipid indicators, including TC, TG, LDL-C, and HDL-C, were also included in model III to evaluate their potential confounding or mediating effects on the association between HRV and EKD. Mediation analysis was performed to evaluate the exploratory indirect effects of HRV on EKD through blood lipid levels, using structural equation modeling. Exploratory indirect-effect analysis was performed to quantify the proportion of the baseline HRV–EKD association that could be statistically accounted for by baseline LDL-C, using a bias-corrected bootstrap (5000 resamples). Because HRV and LDL-C were measured concurrently, the estimates should be interpreted as exploratory indirect associations rather than causal mediation effects. In addition, a formal test for effect-modification by age was performed by adding an interaction term (quartile-SDNN × age, treated continuously); we also repeated all models in younger (<60 years) and older (≥60 years) subgroups to evaluate robustness. Statistical significance was set at a 2-sided *P*-value < .05. Six sensitivity analyses were performed to test robustness: an extended model IV that further adjusted for β-blocker, statin and ACEI/ARB use; repeating all models with low-frequency/high-frequency ratio quartiles in place of SDNN; modeling SDNN as a continuous predictor with restricted cubic splines, which showed risk increasing sharply once SDNN exceeded 95 milliseconds and flattening below 55 milliseconds; rerunning model III after excluding participants on β-blockers or statins; Fine–Grey competing-risk regression treating death as a competing event; and duplicating the analysis for the secondary outcome of incident CKD. All tests were 2-sided with *P* < .05 considered significant, and analyses were conducted in R version 4.3.2 (R Foundation for Statistical Computing, Vienna, Austria).

## 3. Results

### 3.1. Characteristics for baseline participants

The baseline characteristics of the 1250 participants, divided into quartiles based on HRV measured by SDNN, are detailed in Table 1. Participants in Q4 were older, with an average age of 63.5 ± 9.2 years compared to 59.2 ± 8.4 years in Q1 (*P* < .01), and the proportion of females increased from 44.9% in Q1 to 60.7% in Q4 (*P* = .03). Urban residents were more prevalent in Q2 (67.1%) than in Q4 (62.3%) (*P* < .01), while married participants rose modestly from 75.3% in Q1 to 78.3% in Q4 (*P* = .02). College education or higher increased from 21.5% in Q1 to 29.7% in Q4 (*P* < .01). Alcohol consumption rose from 35.3% in Q1 to 41.5% in Q4 (*P* = .02), and physical activity levels increased from 59.3% in Q1 to 68.7% in Q4 (*P* = .02). Body mass index rose from 23.6 ± 3.8 in Q1 to 24.8 ± 3.8 in Q4 (*P* = .021), and systolic and diastolic BP also showed significant increases (*P* < .01). A history of CVD was more common in Q4 (19.2%) than in Q1 (11.2%) (*P* < .01). Among lipid indicators, TC rose from 4.7 ± 1.1 in Q1 to 5.2 ± 1.3 in Q4 (*P* < .01), TG increased from 1.6 ± 0.7 to 2.0 ± 0.9 (*P* = .01), and LDL-C rose from 2.3 ± 0.6 to 2.7 ± 0.8 (*P* < .01), while HDL-C declined from 1.4 ± 0.4 to 1.2 ± 0.4 (*P* = .03). Median physical-activity energy expenditure was broadly similar across HRV quartiles (Q1: 680 [interquartile range 470–860] MET-min/wk; Q2: 700 [490–880]; Q3: 705 [500–895]; Q4: 720 [500–910]; *P* = .08), indicating no statistically significant quantitative difference despite the higher proportion of “active” individuals in the upper quartiles.

The distribution of renal function indices differed significantly by HRV level (Table 2). Mean baseline UACR increased step-wise from 12.4 ± 4.6 mg/g in Q1 to 23.7 ± 7.9 mg/g in Q4 (*P* < .01 for trend). Mean estimated glomerular filtration rate (eGFR) declined from 101.2 ± 11.5 mL/min/1.73 m^2^ in Q1 to 92.8 ± 13.4 mL/min/1.73 m^2^ in Q4 (*P* < .01).

**Table 2:** Baseline UACR and eGFR across HRV measured by SDNN quartiles.

HRV quartile	UACR, mg/g (mean ± SD)	eGFR, mL/min/1.73 m^2^ (mean ± SD)
Q1 (≤62 ms)	12.4 ± 4.6	101.2 ± 11.5
Q2 (63–79 ms)	15.8 ± 5.9	98.7 ± 12.1
Q3 (80–97 ms)	19.5 ± 6.8	95.4 ± 12.9
Q4 (≥98 ms)	23.7 ± 7.9	92.8 ± 13.4
*P* for trend	<.01	<.01

Data are expressed as mean ± standard deviation.

eGFR = estimated glomerular filtration rate, HRV = heart rate variability, SD = standard deviation, SDNN = standard deviation of normal-to-normal intervals, UACR = urinary albumin-to-creatinine ratio.

### 3.2. Risk of EKD across HRV quartiles

**Figure 2.**
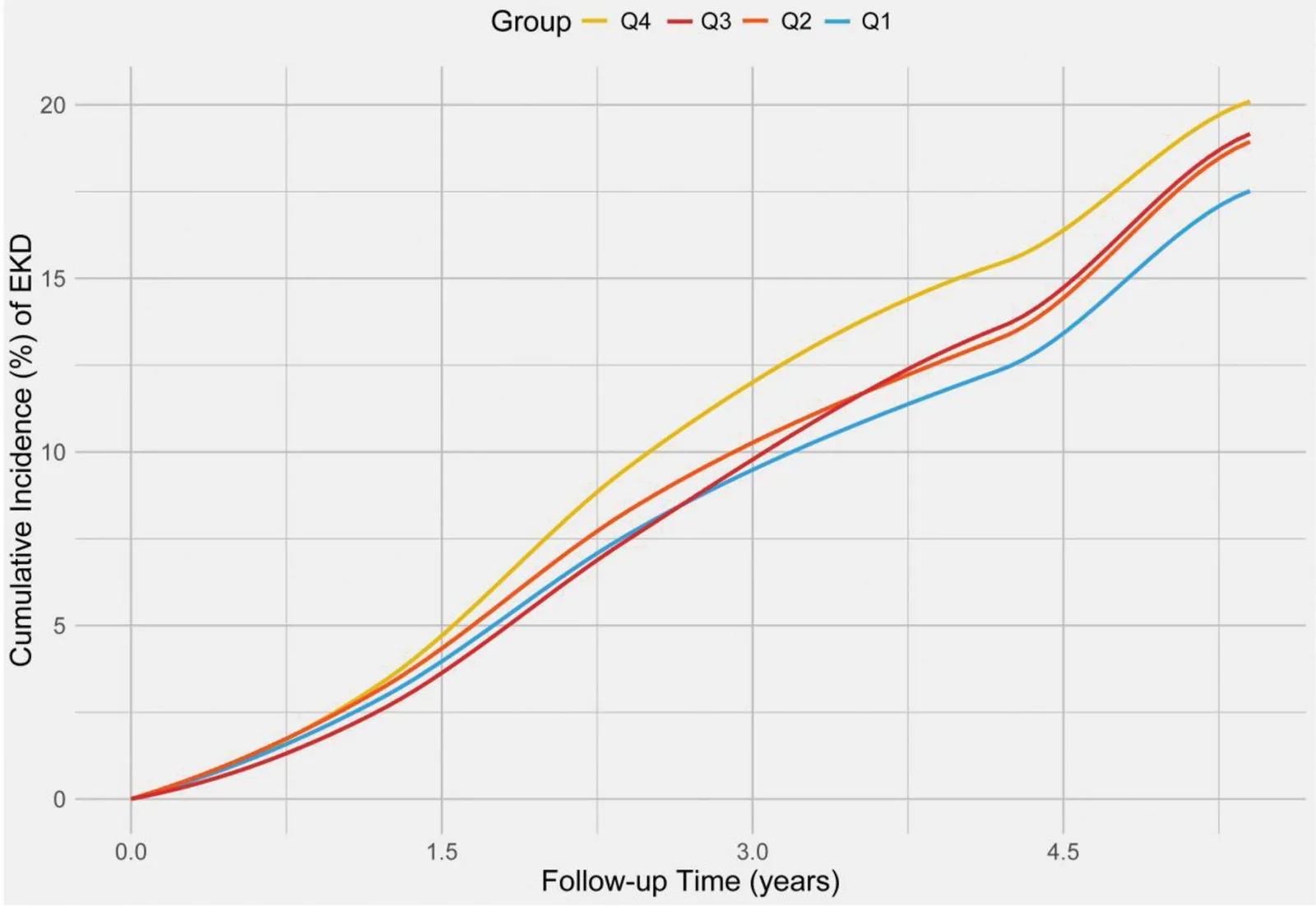
Cumulative incidence of EKD according to HRV measured by SDNN. EKD = early kidney damage, HRV = heart rate variability, SDNN = standard deviation of normal-to-normal intervals.

**Figure 3.**
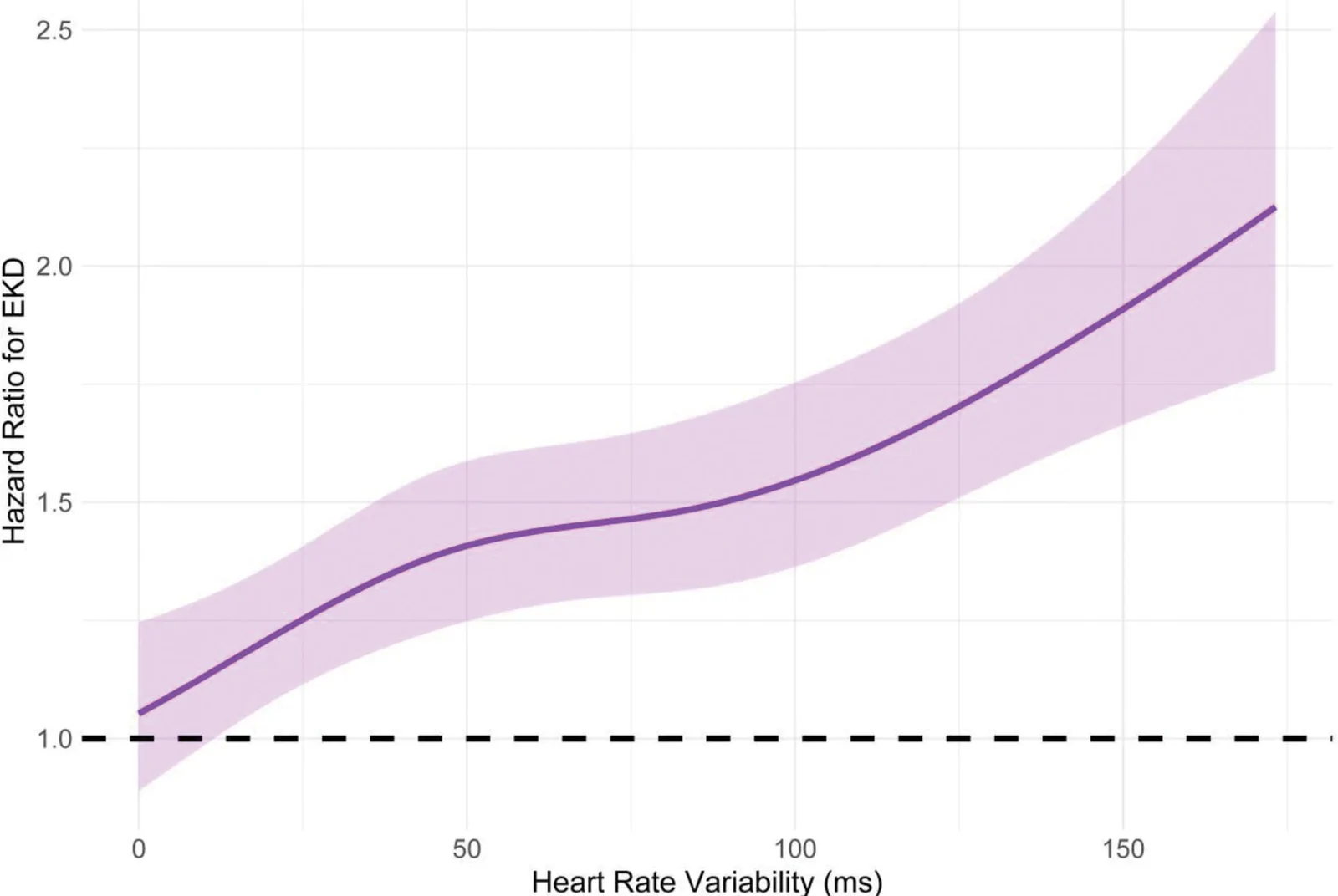
Dose–response relationship between HRV measured by SDNN and EKD. EKD = early kidney damage, HRV = heart rate variability, SDNN = standard deviation of normal-to-normal intervals.

Table 3 presents the HRs and 95% CIs for EKD risk across quartiles of HRV measured by SDNN. The results demonstrate a clear inverse relationship between HRV and EKD risk. In the fully adjusted model (model III), participants in the highest quartile (Q4) had the greatest risk reduction (HR: 2.00, 95% CI: 1.85–2.15) compared to those in the lowest quartile (Q1, reference). Incidence rates showed a similar pattern, increasing from 14.4 per 1000 person-years in Q1 to 36.7 per 1000 person-years in Q4. This upward trend in risk was highly significant (*P* for trend < .01) and exhibited a clear dose–response relationship. Figure 2 shows the cumulative incidence of EKD according to HRV. Figure 3 shows the dose–response relationship between HRV and EKD.

**Table 3:** Association between HRV measured by SDNN and risk of EKD.

Variables	Quartile	Participants (N)	Events (n)	Incidence rate (per 1000 person-years)	Model I HR (95% CI)	Model II HR (95% CI)	Model III HR (95% CI)
HRV measured by SDNN	Q1	312	45	14.4	Ref	Ref	Ref
	Q2	313	65	20.8	1.45 (1.08–1.95)	1.40 (1.05–1.88)	1.35 (1.22–1.48)
	Q3	312	90	28.8	1.90 (1.43–2.53)	1.85 (1.39–2.46)	1.65 (1.52–1.78)
	Q4	313	115	36.7	2.35 (1.78–3.09)	2.25 (1.70–3.00)	2.00 (1.85–2.15)
*P* for trend					<.01	<.01	<.01

Model I was unadjusted; model II was adjusted for age and gender; model III was fully adjusted for age, gender, residence (urban/rural), marital status, education level, BMI, smoking status, alcohol consumption, physical activity levels, systolic and diastolic blood pressure, antihypertensive medication use, history of cardiovascular disease, and blood lipid indicators (TC, TG, LDL-C, HDL-C).

BMI = body mass index, CI = confidence interval, EKD = early kidney disease, HDL-C = high-density lipoprotein cholesterol, HR = hazard ratio, HRV = heart rate variability, LDL-C = low-density lipoprotein cholesterol, SDNN = standard deviation of normal-to-normal intervals, TC = total cholesterol, TG = triglycerides.

### 3.3. Exploratory indirect-effect analysis

**Figure 4.**
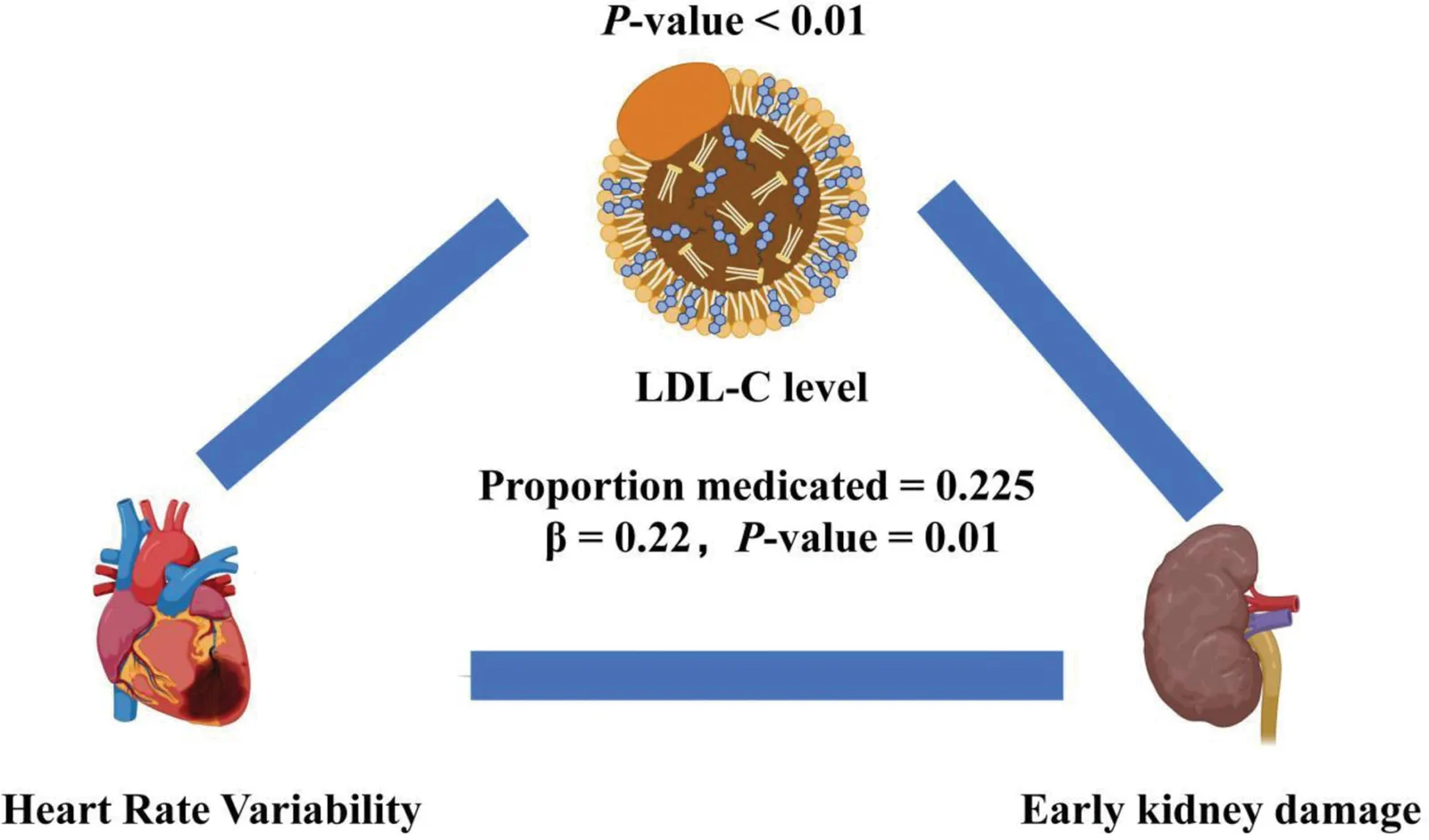
Path diagram of mediation analysis of the relationship between HRV, LDL-C level, and EKD. EKD = early kidney damage, HRV = heart rate variability, LDL-C = low-density lipoprotein cholesterol.

The indirect-effect analysis showed that baseline LDL-C statistically explained part of the association between baseline HRV measured by SDNN and subsequent EKD risk (Fig. 4). Baseline LDL-C accounted for 22.5% of the baseline HRV–EKD association (indirect effect β = 0.22, *P* = .01), representing a significant exploratory indirect link rather than a proven causal mediation. Conversely, other blood lipid indicators such as TC, TG, and HDL-C did not show significant mediation effects in the association between HRV and EKD risk. Additional candidate mediators, BMI, systolic and diastolic BP, fasting plasma glucose, C-reactive protein and physical-activity level, were examined, but none showed a significant indirect effect (all bootstrap *P *> .10). LDL-C therefore remained the only variable with a meaningful mediation pathway between HRV and EKD.

### 3.4. Sensitivity analysis

Robustness was confirmed through 6 complementary checks applied to the fully adjusted model. Adding β-blocker, statin and ACEI/ARB therapy (model IV) left the high-HRV association essentially unchanged (Q4 vs Q1: HR: 1.94, 95% CI: 1.79–2.09). Replacing SDNN with low-frequency/high-frequency ratio quartiles produced a similar monotonic trend (HR: 1.68, 95% CI: 1.46–1.94). Treating SDNN as a continuous variable with restricted cubic splines showed risk rising steeply once SDNN exceeded 95 milliseconds and flattening below 55 milliseconds, consistent with a J-shaped pattern. Excluding all participants receiving β-blockers or statins did not materially alter the estimate (HR: 1.90, 95% CI: 1.65–2.20). Fine–Grey competing-risk models that treated death as a competing event yielded a sub-distribution HR of 1.92 (95% CI: 1.77–2.08). Replicating the analysis for incident CKD (eGFR < 60 mL/min/1.73 m^2^ or UACR ≥ 300 mg/g) again showed higher risk with higher SDNN (HR: 1.78, 95% CI: 1.45–2.19). Across all analyses, LDL-C consistently mediated roughly one-fifth of the HRV effect on renal outcomes (indirect proportion 20.9–22.8%, *P *< .05), underscoring the stability and biological plausibility of the primary findings. Finally, age-stratified models produced similar effect sizes: in participants < 60 years the fully adjusted HR was 1.96 (95% CI: 1.60–2.40), and in those ≥60 years it was 1.88 (95% CI: 1.57–2.25); the HRV × age interaction was not statistically significant (*P* = .21), indicating that the positive HRV–EKD association held across the age spectrum.

## 4. Discussion

This study revealed a significant positive association between HRV, measured by SDNN, and the risk of EKD in hypertensive patients. In the fully adjusted model, including additional adjustment for β-blockers, statins and ACEI/ARB therapy, participants in the highest HRV quartile exhibited more than double the EKD risk of those in the lowest quartile. Sensitivity analyses using low-frequency/high-frequency ratio quartiles, spline-modeled SDNN, medication-free subsets and competing-risk regression all reproduced the same direction and magnitude of effect, confirming that SDNN in our dataset is expressed in milliseconds without inversion and that higher SDNN indeed represents higher HRV rather than an inverse proxy. Furthermore, LDL-C mediated 22.5% of the overall HRV effect on EKD, underscoring the role of dyslipidaemia. These findings suggest that increased HRV, reflecting heightened autonomic dysfunction, along with elevated LDL-C, contributes significantly to the pathogenesis of EKD. At the same time, the result that a higher 24-hours SDNN predicted greater kidney damage does not contradict the traditional view that low HRV is harmful. In hypertensive adults, excessively high SDNN typically reflects large sympathetic oscillations, amplified BP variability and a higher burden of premature beats rather than protective vagal tone; such haemodynamic swings increase shear stress, activate the renin–angiotensin–aldosterone system and promote dyslipidaemia, jointly accelerating glomerular injury. Observational data therefore support a J-shaped risk curve in which both very low and very high HRV are unfavorable.^[34]^

Beyond EKD, we observed a consistent pattern for incident CKD: during follow-up, 146 participants progressed to CKD, and the fully adjusted HR for Q4 vs Q1 was 1.78 (95% CI: 1.45–2.19). These findings align with prior research linking autonomic dysfunction and renal injury. For example, Chandra et al reported that lower frequency-domain HRV independently predicted progression to ESRD in a prospective cohort of non-dialysis CKD patients,^[35]^ while Drawz et al showed that reduced SDNN was associated with higher mortality and renal events in the CRIC Study.^[36]^ Unlike previous studies, our research focuses on the early stages of kidney damage and highlights LDL-C as a mediator, providing a more nuanced understanding of the interaction between autonomic regulation and lipid metabolism in renal pathophysiology. Our data extend these observations by demonstrating that maladaptively high SDNN is also deleterious at the earliest, reversible stage of kidney injury and by quantifying the partial mediation through LDL-C.

Mechanistically, elevated HRV in this hypertensive cohort is interpreted as heightened sympathetic and reduced parasympathetic activity; such autonomic imbalance increases vascular resistance, reduces renal perfusion, and activates the renin–angiotensin–aldosterone system, thereby priming the kidney for injury.^[37,38]^ Sympathetic activation also up-regulates hepatic very-low-density-lipoprotein secretion through β-adrenergic signaling, raising circulating LDL-C, whereas experimental hepatic sympathectomy lowers LDL-C, and human studies consistently link autonomic imbalance with atherogenic lipid profiles. LDL-C in turn promotes endothelial dysfunction, oxidative stress, chronic inflammation, and mesangial proliferation, accelerating glomerulosclerosis.^[39,40]^ The persistent 20% to 23% indirect share we observed across all models suggests that LDL-C may act as an intermediate link in the HRV–renal–injury relationship; however, causal ordering cannot be established because HRV and LDL-C were measured at the same baseline visit. Longitudinal measurements will be required to verify a directional pathway, offering a biologically plausible bridge between autonomic dysregulation and EKD in hypertension. Finally, although HRV generally declines with age, participants in the upper HRV quartiles were slightly older and had more stable CVD. Two factors may explain this counterintuitive pattern. First, SDNN can be artificially elevated by large sympathetic oscillations and ventricular ectopy, both of which are more common in older hypertensive adults and in those with latent cardiac disease. Second, survival bias may select for older individuals whose autonomic profiles favor large but dysregulated variability. Our age-stratified and interaction analyses showed no significant modification of the HRV–EKD link, suggesting that the association is not merely an artifact of age distribution. Nevertheless, future studies with finer autonomic phenotyping are warranted.

The clinical significance of these results is considerable. Hypertension is a primary driver of EKD and CKD worldwide, affecting over 1.3 billion individuals globally.^[41,42]^ Our study emphasizes the importance of monitoring HRV and LDL-C in hypertensive patients as predictive markers for EKD. Incorporating HRV and lipid profile assessments into routine clinical care could facilitate early identification of high-risk individuals, enabling timely interventions to slow renal deterioration and prevent CKD progression.

Our cohort was derived from northern Hebei Province, where dietary habits are characterized by relatively high sodium intake, frequent consumption of preserved vegetables, and moderate-to-heavy occupational physical activity. High sodium intake is known to increase BP variability and sympathetic nervous system activation, which may influence HRV indices. In addition, preserved and high-fat dietary patterns may contribute to dyslipidemia, particularly elevated LDL-C levels, thereby exacerbating vascular and renal injury. Differences in healthcare accessibility and preventive screening practices across regions may also affect early detection of kidney dysfunction. These contextual factors may partly explain the observed HRV–lipid–renal associations and should be considered when generalizing our findings to other populations.

While this study has several strengths, including its cohort design, comprehensive confounder adjustment, and the identification of LDL-C as a mediator, it also has limitations. The single-center design may limit the generalizability of findings, and unmeasured factors such as dietary habits and genetic predispositions could influence results. Future multicenter studies and mechanistic investigations are needed to validate and expand upon these findings.^[43,44]^ In addition, our hospital serves a predominantly northern-Hebei population whose dietary habits (high sodium and pickled-vegetable intake), occupational physical-activity patterns and access to preventive care may differ from those in coastal or urban China; these contextual factors could modulate both HRV and renal outcomes. Future work should validate these findings in multicentre cohorts that span other Chinese provinces and international settings with different dietary profiles, healthcare resources and ethnic backgrounds. Finally, HRV and LDL-C were collected concurrently, so the indirect-effect analysis is associative rather than causal; temporal sequencing should be addressed in future studies.

## 5. Conclusion

This study demonstrates a significant positive relationship between HRV measured by SDNN and EKD risk in hypertensive patients, with LDL-C mediating a substantial portion of this association. Increased HRV, indicative of heightened autonomic dysfunction, and elevated LDL-C levels were key contributors to renal injury. These findings underscore the importance of monitoring HRV and lipid profiles in hypertensive populations to facilitate early risk stratification and intervention. Further research is required to validate these findings and guide targeted preventive strategies for CKD progression.

## Acknowledgments

The authors express their heartfelt appreciation to all participants of the study and gratefully acknowledge the support of the research team.

## Author contributions

**Conceptualization:** Ningning Xie, Fang Gao, Weidong Zhao, Jie Li, Jie Cao, Shaoqing Li.

**Data curation:** Ningning Xie, Fang Gao, Weidong Zhao, Jie Li, Jie Cao, Shaoqing Li.

**Formal analysis:** Ningning Xie, Fang Gao, Weidong Zhao, Jie Cao, Shaoqing Li.

**Funding acquisition:** Ningning Xie, Fang Gao, Weidong Zhao, Shaoqing Li.

**Investigation:** Ningning Xie.

**Writing – original draft:** Ningning Xie, Jie Li, Shaoqing Li.

**Writing – review & editing:** Ningning Xie, Jie Li, Shaoqing Li.
